# Why We Do Not Recommend That People With High‐Risk Smoldering Myeloma Receive Treatment

**DOI:** 10.1002/ajh.70159

**Published:** 2025-12-04

**Authors:** Ghulam Rehman Mohyuddin, Mackenzie Lemieux, Rajshekhar Chakraborty, Morie Gertz

**Affiliations:** ^1^ Division of Hematology and Hematological Malignancies, Huntsman Cancer Institute University of Utah Salt Lake City Utah USA; ^2^ Division of Internal Medicine University of Utah Salt Lake City Utah USA; ^3^ Division of Hematology/Oncology Columbia University Irving Medical Center New York City New York USA; ^4^ Division of Hematology Mayo Clinic Rochester Minnesota USA

## 1 Introduction

Smoldering myeloma (SMM) affects approximately 1 in 200 individuals over age 40 and represents a precursor state to multiple myeloma [1]. The 2014 diagnostic reclassifications fundamentally altered SMM's natural history by moving the highest‐risk patients into the myeloma category, while advances in imaging—particularly MRI and PET—now detect lesions that would have been missed by conventional radiography [2, 3]. These dual changes have rendered previous natural history studies and risk stratification systems obsolete. Notably in 2025, both the European Commission and the United States FDA approved the first ever treatment of high‐risk SMM based on the Phase III AQUILA trial [4]. While recent trials have demonstrated statistically significant improvements in progression‐free survival (PFS) with early intervention, we remain convinced that high‐risk SMM should not be routinely treated.

## 2 Risk Stratification Systems are Imperfect

Identifying patients who might benefit from SMM treatment is still an unsolved problem. A frequently quoted study to describe the natural history of SMM (10% risk of progression in the first 5 years) included patients who were diagnosed between 1970 and 1995, much before the advent of advanced imaging and diagnostic reclassifications [5]. Several other risk stratification systems have been developed to try to identify these patients and guide disease management. These too have the limitation of inclusion of patients that have not undergone rigorous advanced imaging with MRI or PET and being derived from datasets prior to diagnostic reclassifications [6]. There is discordance between different criteria, with a study showing that the overall agreement between three models of SMM risk assessment (Old Mayo, IMWG 20/2/20 and PETHEMA) is only 16.6% [6]. A recent retrospective cohort study in which patients underwent advanced imaging also demonstrates that these conventional staging systems overestimate the risk of progression [7]. As a result, our current risk stratification systems fail to predict who progresses to myeloma, who will experience true morbidity at progression, and who may benefit from early intervention.

## 3 Our Interventions Prevent Asymptomatic Laboratory and Imaging Abnormalities, Not Morbidity

Previous SMM randomized trials did not provide sufficient insights on what progression events were being prevented by therapy. It was thus unknown whether treatment was preventing laboratory abnormalities, or morbidity. The recent AQUILA trial, which was a randomized comparison of observation versus daratumumab for high‐risk SMM provided this information, and the recent FDA Oncological Drugs Advisory Committee (ODAC) offered insights into progression events [8, 9].

In the AQUILA trial, although overall progression events were more frequent in the observation arm compared to the daratumumab arm, these events were largely asymptomatic [8]. Among the 166 patients who experienced progression events across both arms, only three patients developed symptomatic bone pain—and two of these were in the daratumumab‐treated group [8]. All other bone events appeared to be asymptomatic imaging findings. The median hemoglobin drop among patients who developed “anemia progression” was 1.7 g/dL, with no corresponding reports of fatigue, and no anemia severe enough to require a blood transfusion [8].

Most progression events represented asymptomatic findings discovered through intensive surveillance: 16 patients in the control arm versus five in the daratumumab arm had ≥ 60% plasma cells detected only through protocol‐mandated repetitive bone marrow sampling. In the ECOG E3A06 trial exploring lenalidomide versus observation, 76% of the control arm were progression‐free at 2 years, suggesting overtreatment in ostensibly high‐risk patients [4]. This raises a fundamental question: are we treating a disease or treating test results? Three recent retrospective datasets of patients with SMM on observation highlight the low incidence of morbidity [10, 11, 12], and prior data also indicate that progression events in patients with SMM are almost always not morbid [7, 13]. Furthermore, 20% of patients diagnosed with myeloma in the observation arm of the AQUILA trial did not initiate treatment, raising questions about the clinical meaningfulness of progression as currently defined in myeloma—even among investigators treating patients on this trial [8].

## 4 Post‐Progression Treatment Was Inadequate in Control Arms

For trial testing a drug in SMM that is already known to be efficacious in myeloma, the control arm should receive effective therapy incorporating that drug upon progression [14]. Unfortunately, not all SMM randomized trials have met this bar. The Spanish trial demonstrated an overall survival (OS) benefit with the use of lenalidomide plus dexamethasone compared to the observation [15]. However, this trial was not powered for OS analysis, and lenalidomide was received by only 28% of patients in the control arm upon progression to myeloma [15]. As a result, this trial compared giving an effective drug early to one cohort of patients versus not giving an effective drug at all to most patients in the other cohort.

The ECOG trial of lenalidomide versus observation has not yet demonstrated an OS advantage, nor provided details of post‐progression treatment. Given that this trial is being conducted exclusively in the United States, we suspect the lack of OS advantage is due to high‐quality care being provided to patients when they progress to myeloma [16].

The AQUILA trial of daratumumab versus observation was not powered for OS, and hence, any inferences regarding OS must be taken with that context. This was a global trial conducted in areas with widely different access to therapies upon progression to myeloma. Nevertheless, this trial already appears to be demonstrating improved OS with 5‐year OS at 93.0% with daratumumab (versus 86.9% with active surveillance). This trial enrolled from December 2017 through May 2019, but patients in the control arm would have likely progressed in the few years that followed, at a time when daratumumab had shown efficacy for newly diagnosed myeloma. The ALCYONE trial had led to daratumumab approval for newly diagnosed myeloma by the FDA as early as May 2018 [17]. In our opinion, it was a missed opportunity to not have provided daratumumab‐based regimens (or at the very minimum contemporary triplet therapy) to the control arm at progression to myeloma. This is best exemplified by the fact that of 105 patients in the control arm who went on to receive therapy, only 29 (27.8%) received bortezomib/lenalidomide/dexamethasone, and only 18% received a regimen containing anti‐CD38 therapy.

A recent correspondence by the authors of this paper reports that of the 41 deaths on trial, only 12 occurred after the start of initial therapy for progression—nine in the active‐monitoring group and three in the daratumumab group [18]. The cause of death was unknown for many patients on the trial, and there is an existing imbalance in the number of deaths attributed to reasons other than myeloma (12 in the daratumumab arm, 17 in the observation arm). We hypothesize that the differences seen could be due to chance alone, given that the trial was not powered for OS. Another possibility, given the global nature of this trial and widely varying local standards of care, is that inadequate screening could have resulted in a higher likelihood of sudden death due to a missed diagnosis of AL amyloid.

Thus, despite the OS results seen in the AQUILA trial, there is no proof that early intervention improves OS when high‐quality therapy is provided upon diagnosis of myeloma.

## 5 The Financial and Physical Toxicity Is Substantial

Daratumumab treatment for SMM costs approximately $200 000 per patient per year in the United States [19]. There are additional costs of IVIG in patients on daratumumab who develop markedly low IgG. This financial toxicity is felt by the patient and society alike. Beyond financial toxicity, the physical burden is real. Infections of any grade occurred in 80% of patients receiving daratumumab compared to 45% in the observation cohort, while Grade 3 or higher infections occurred in 17% versus 5% of patients, respectively. More patients receiving daratumumab experienced treatment emergent adverse events (97% vs. 83%). All‐grade fatigue was twice as prevalent in the daratumumab arm (42% vs. 21%), which is all the more concerning given that it was being administered in an asymptomatic population, and that the prevention of anemia as a CRAB event is intuitively thought of as improving fatigue [8].

Other SMM interventions have been substantially more toxic. The GEM‐CESAR trial evaluated a prolonged course of carfilzomib, lenalidomide, dexamethasone and incorporated a stem cell transplant for patients with SMM. In this trial, 4.4% of patients (4 of 90) died due to reasons unrelated to myeloma progression—causes such as stroke, cardiac arrest, lung cancer, and myelodysplastic syndrome [20]. Without concurrent control groups to establish baseline mortality and competing risks in similar patient populations, it is unknown if patients would have lived longer had they been observed or treated with gentler approaches.

## 6 Modern Surveillance Strategies Are Highly Effective

The premise of early intervention rests on preventing “irreversible” organ damage. However, contemporary surveillance with advanced imaging makes this argument obsolete. Whole‐body diffusion‐weighted MRI can detect focal lesions with far greater sensitivity than the PET/CT or low‐dose CT used in AQUILA [21, 22, 23].

Most importantly, regular monitoring allows intervention before symptomatic complications develop, as is being prospectively evaluated in the SPOTLIGHT trial (NCT06212323). In contemporary cohorts using modern imaging, morbid progression events are remarkably rare [10, 11, 24]. When symptomatic events do occur, they are typically detected early enough that permanent organ dysfunction is uncommon.

## 7 The Biological Rationale Is Questionable

Treating SMM is based on the premise that if curative treatment exists, a patient with a lower burden of disease (i.e., SMM) may have a higher chance of being cured than a patient with symptomatic myeloma. It is thus commonly assumed that SMM is more responsive to treatment than myeloma is [25]. A recent systematic review showed that responses were no better or deeper in SMM than myeloma when comparing response rates across all studied regimens [26]. In a retrospective cohort study, patients that were asymptomatic but received therapy for SLiM criteria did not have higher complete remission or measurable residual disease negativity rates, further suggesting earlier intervention does not deepen response [27].

The GEM‐CESAR trial further confirms this hypothesis. Despite subjecting patients to intensive therapy with carfilzomib, lenalidomide, dexamethasone, transplant and prolonged maintenance, only 28% (25 out of 90 patients) remained MRD negative at 10^−5^ 4 years after transplant in GEM‐CESAR when analyzed on an intent to treat basis [20]. It is likely that an even lower number of patients were MRD negative at lower thresholds. Even with such intensive therapies, a cure was not obtained for the vast majority of patients with SMM in this trial.

Even with daratumumab in the AQUILA trial, the progression‐free survival at 5 years was 63.1%, indicating that some patients with the highest risk SMM still progressed to myeloma, and now were daratumumab refractory at the time of diagnosis of myeloma, reducing the number of options available for their treatment.

## 8 How We Counsel Our Patients

When counseling patients with newly diagnosed SMM, we explain that modern quadruplet regimens achieve deep responses in most patients. The question is not whether we can delay laboratory progression—clearly, we can—but whether doing so improves outcomes that matter to patients. We explain to patients that the risk of meaningful morbidity with modern surveillance is very low, likely less than 5% over the first several years. We explain that treating SMM means accepting certain toxicity and cost, with a lack of survival benefits. Most importantly, we emphasize that if they do develop myeloma, we have excellent treatments available.

Using a shared decision‐making approach, we acknowledge that some clinicians do consider treatment of SMM. A recent qualitative assessment of patient decision‐making in myeloma showed that patients have increased hope when they learn about new and available treatments, even if just offered on an as‐needed basis [28]. We discuss that daratumumab appears safer than lenalidomide and can be considered in the broader context of the individual patient's financial, family, and other social factors. We also recommend treatment for SMM that is clearly progressing and evolving to myeloma, and advocate for treatment before end‐organ damage occurs in this context.

## 9 Conclusions and Future Directions

The statistical significance achieved in SMM trials should not obscure the lack of meaningful clinical benefit seen in those trials. In our opinion, we are preventing asymptomatic laboratory changes, not meaningful morbidity. We are treating our anxiety about “doing nothing” rather than treating a disease that threatens patients.

The decision to intervene based on a single data set at one point in time is problematic. Future research should focus on exploring ways to use dynamic monitoring of laboratory changes and imaging to track the trajectory of evolution towards symptomatic myeloma, such as is being done in the SPOTLIGHT study (NCT06212323). Recent work has defined genomic myeloma characteristics in those with SMM, which has modestly improved the prediction value of the 2/20/20 model (from a c‐index of 0.74–0.79) [29]. These patients can be tested in future approaches assessing the benefits of early intervention. There are many hematological cancers that do not need treatment despite being cancer genomically, and whether early intervention will improve survival, or prevent morbidity, for this patient population should be explored in future studies.

Until trials demonstrate clear OS benefits with appropriate post‐protocol therapy, we will continue to recommend active surveillance for patients with SMM. The burden of proof for treating asymptomatic patients should be high. Current evidence for SMM treatment has not met that burden.

## Conflicts of Interest

The authors declare no conflicts of interest.

## Data Availability

Data sharing not applicable to this article as no datasets were generated or analyzed during the current study.
